# Modeling growth curves to estimate the suitable slaughter age for the ostriches (*Struthio camelus*)

**DOI:** 10.1007/s11250-025-04452-1

**Published:** 2025-04-30

**Authors:** Ha Xuan Bo, Han Quang Hanh, Do Thi Hue, Dang Vu Hoa, Nguyen Thi Hoa, Nguyen Thanh Cong, Nguyen Thi Vinh, Dang Thuy Nhung, Do Duc Luc

**Affiliations:** 1 Faculty of Animal Science, Vietnam National University of Agriculture, Hanoi, Vietnam; 2 Davies Livestock Research Centre, School of Animal & Veterinary Sciences, University of Adelaide, Roseworthy Campus, Roseworthy, SA 5371 Australia; 3 National Institute of Animal Sciences, Hanoi, Vietnam

**Keywords:** Bodyweight, Growth performance, Mathematical function, Ostrich, Slaughter age

## Abstract

Using an accurate growth curve to estimate the suitable age for slaughter has many benefits for ostrich farming. This study was carried out to compare eight growth curve models for estimating body weight and monthly gain of ostriches raised in tropical conditions and then suggested the suitable slaughter age for these ostriches. Two hundred eighty-two ostriches (124 males and 158 females) were raised from birth to 12 months of age to measure individual monthly body weight, and the growth data was analyzed by R software. Eight growth functions (von Bertalanffy, Bridges, Janoschek, Gompertz, Logistic, Lopez, Richards, and Weibull) were used to create the growth curves of ostrich. The Weibull function was the best model among 8 tested models to describe the growth of both male and female ostriches, and the monthly gain (MG) reached the maximum at the 6^th^ month of age. The maximum average monthly gain (AMG) was obtained between the 9^th^ and 10^th^ month of age for both males and females. The best-fit model in this study (Weibull function) suggests the suitable slaughter ages were estimated from the 9^th^ to 10^th^ month of age for both male and female ostriches, with estimated body weights of 93 kg for male and 90 kg for female.

## Introduction

The ostrich (*Struthio camelus*) is the largest, non-flying bird, classified as class Aves, order Struthioniformes, family Struthionidae, genus Struthio, and species S. camelus (Cooper et al. 2009; Kummrow 2015; Schmidt-Nielsen et al. 1969). Ostriches produce many different useful products for human demand such as meat, eggs, feathers, and leather (de Rezende et al. 2021; Ghavi Hossein-Zadeh 2024). Ostriches produce higher meat and egg products compared to other poultry species, because the maximum body weight of ostriches is from 120 to 160 kg, and hens can produce about 100 huge eggs per year (Kummrow 2015). In addition, ostrich is well-adapted to different ecological regions, and good at forage sources utilization to produce meat, eggs, skins, and feathers (Kummrow 2015). Thus, ostriches contribute to develop the rural economic, food security and job creation in many regions (Ghavi Hossein-Zadeh 2024).

To achieve maximum profit in ostrich farming, it is essential to understand the ostrich’s growth performance, nutritional needs and other environmental factors. Understanding the growth curve of ostriches not only helps to forecast the ostrich’s growth at each stage of age but also formulates the feeding regime and determines the appropriate age to slaughter. Thus, it contributes to developing ostrich productivity and achieves higher production efficiency.

Ostriches are increasingly adopted in many countries around the world, including Vietnam. The body weight of fattening ostriches raised in Vietnam at 12 months of age was high (about 96 to 100 kg per bird), and a female ostrich can produce 2.5 - 3.0 tons of meat a year, which is slightly higher than other species such as chicken, ducks and goats in Vietnam (Tien et al. 2007). The ostrich’s growth curve has been investigated by some researchers in Spain and Brazil (de Rezende et al. 2021; Faridi et al. 2015; Ramos et al. 2013) to predict the body weight at different growth stages, however, the growth curve of ostrich has not yet studied in Vietnam. Although there are some studies reported the growth curves of other livestock species raised in Vietnam such as chicken (Bo et al. 2022; Bo and Nhung 2022; Tuan et al. 2022), duck (Thinh et al. 2021), pig (Bo et al. 2020; Bo et al. 2023), and cattle (Vinh et al. 2020), but studies on modeling the growth curve of ostrich in Vietnam have not investigated. Thus, it is important to create an accurate model to predict the growth of the ostrich raised in Vietnam. The result of this study not only contributes to developing ostrich in Vietnam but also provides an accurate tool for ostrich farming management in other countries, which have similar conditions as Vietnam.

In this study, eight non-linear models (von Bertalanffy, Bridges, Janoschek, Gompertz, Logistic, Lopez, Richards, and Weibull) were utilized to create the growth curve of fattening ostriches raised in Vietnam, and then the better model was suggested. In addition, the body weight, monthly weight gain, and suitable slaughter age for fattening ostriches were also predicted using the best model among the tested models herein to provide the ostrich producers with technical recommendations to achieve the genetic potential of ostrich.

## Materials and methods

### Animals, diets, and experimental design

Ostriches in this study were raised at the Ostrich Breeding Research Station, Thuy Phuong Poultry Research Center, Institute of Animal Science, in Ba Vi district, Hanoi, Vietnam, from July 2018 to December 2022. Ba Vi district is located in the North-West of Hanoi capital, and it has four seasons (spring, summer, autumn, and winter) (GSO 2019). The average annual temperature is about 23.3^o^ C and the total number of hours of sunshine per year is about 1,514 hours (GSO 2019).

Two hundred eighty-two ostriches (124 males and 158 females) at birth were tagged individually on their heads and necks. All ostriches were raised in the same pen and grazed in the same yard area under non-limiting conditions for genetic potential growth. The mixed diet that included a combination of forage and commercial compound feed was used for *ad libitum* feeding. The forage (including water spinach, elephant grass, and banana tree) was used at 20% in the diet, and commercial feed was used at 80% in the diet for all ostriches, but the ingredients proportion of commercial feed was varied depending on their age. The commercial feed of ostriches from 1 to 4 months of age included 21% crude protein, 2,900 kcal/kg metabolizable energy, and 5% fiber, from 4 to 8 months of age was 18% crude protein, 2,600 kcal/kg metabolizable energy, and 8% fiber, and from 9 to 12 months of age was 16% crude protein, 2,500 kcal/kg metabolizable energy, and 10% fiber. Ostriches were fed twice per day, at 7h30 and 14h00.

### Measurement and statistical analyses

The body weight (BW) of an individual ostrich was measured at birth and every month until 12 months of age by using an electronic weighing scale (Mettler Toledo model). The growth data were analyzed in R software (R version 4.2.2, https://cran.r-project.org/bin/windows/base/old/4.2.2/). Eight mathematical functions, namely von Bertalanffy, Bridges, Janochek, Gompertz, Logistic, Lopez, Richards, and Weibull (Bridges et al. 1992; Gompertz 1825; Lopez et al. 2000; Murthy et al. 2004; Pearl 1977; Richards and Kavanagh 1945; Von Bertalanffy 1957) were used to describe growth performance of male and female ostriches separately. The formular of eight non-linear growth functions for estimating BW at certain time t (BW_t_), age at inflection, and BW at inflection was given in Table 1.

**Table 1:** The formula of eight models for estimating body weight (BWt), age at inflection, and body weight at inflection

No	Functions	Equation	Age at inflection	Weight of inflection
1	von Bertalanffy	$BWt=\alpha \times \left(1-\beta \times {e}^{-k{t}^{3}}\right)$	$\frac{\text{ln}\left(3\times \beta \right)}{k}$	$\frac{8\times \alpha }{27}$
2	Bridges	$BWt=B{W}_{0}+\alpha \times \left(1-{e}^{-k{t}^{m}}\right)$	-	-
3	Janoschek	$BWt=\alpha -\left(\alpha -B{W}_{0}\right)\times {e}^{-k{t}^{m}}$	$\frac{\left(m-1\right)}{{\left(k\,\times\, m\right)}^\frac{1}{m}}$	$\alpha -\left(\alpha -k\right)\times {e}^{\frac{-\left(m-1\right)}{m}}$
4	Gompertz	$BWt=\alpha \times {e}^{-\beta \,\times\, {e}^{-kt}}$	$\frac{\text{ln}\left(\beta \right)}{k}$	$\frac{\alpha }{e}$
5	Logistic	$BWt=\frac{a}{\beta\, \times\, \left(1\,+\,{e}^{-kt}\right)}$	$\frac{\text{ln}\left(\beta \right)}{k}$	$\frac{\alpha }{2}$
6	Lopez	$BWt=\frac{\left(B{W}_{0}\times\, {\beta }^{k}+\alpha \,\times\, {t}^{k}\right)}{\left({\beta }^{k}+{t}^{k}\right)}$	$\beta \times {\left[\frac{\left(k-1\right)}{\left(k+1\right)}\right]}^\frac{1}{k}$	$\frac{\left[\left(1+\frac{1}{k}\right)\times B{W}_{0}+\left(1-\frac{1}{k}\right)\times a\right]}{2}$
7	Richards	$BWt=\frac{\alpha }{{\left(1-\beta\, \times\, {e}^{-kt}\right)}^\frac{1}{m}}$	$\frac{\text{ln}\left(m\times \beta \right)}{k}$	$\frac{a}{{\left(m+1\right)}^\frac{1}{m}}$
8	Weibull	$B{W}_{t}=\alpha -\beta \times {e}^{-k{t}^{m}}$	-	-

BWt = body weight (kg) at the time t; BW0 = initial body weight (kg); α = upper asymptotic body weight (kg); t = age (months);

β, k, and m = parameters specific for the function; β characterizes the first part of growth before the point of inflection; k describes the second part in which growth rate decreases until the animal reaches the upper asymptotic body weight or mature body weight (α), m is the shape parameter determining the position of the curve point, e = the Euler’s number (~ 2.718282).

The mathematical models for each gender were fitted using the nlsLM() function in the minpack.lm package in R software (Elzhov et al. 2016). Different parameters of growth functions were also estimated for ostriches, including α, β, k and m (Table 2). The α is the upper asymptotic body weight (kg), β characterizes the first part of growth before the point of inflection, k describes the second part in which the growth rate decreases until the animal reaches the upper asymptotic body weight, and m is the shape parameter determining the position of the curve point. In addition, some parameters that are essential for comparing model performance were generated in R software, including Akaike’s information criterion (AIC), Bayesian information criterion (BIC), standard error of the regression (SER), and coefficient of determination (goodness-of-fit, R^2^) (Table 3). The comparison between growth functions was based on the goodness-of-fit (R^2^) to the individual growth curves of two data sets (males and females ostriches) (0 ≤ R^2^ ≤ 1), higher R^2^ indicated the better fit (Ramos et al. 2013). In addition, the BIC and the AIC parameters were used for identifying the best model (Bo et al. 2023; Chakrabarti and Ghosh 2011; Ramos et al. 2013). The best model among tested models was confirmed when the parameters of AIC, BIC, and SER were the lowest, and the coefficient of determination (R^2^) was the highest (Bo et al. 2023; Ramos et al. 2013).

**Table 2:** Estimated parameters of growth functions for ostriches

Functions	Sex	α (kg)	β	k	m	BW_0_ (kg)
von Bertalanffy	Male	137.95 ± 1.47	0.99 ± 0.013	0.22 ± 0.004	-	-
	Female	136.58 ± 1.48	0.97 ± 0.011	0.21 ± 0.004	-	-
Bridges	Male	111.64 ± 0.97	-	0.01 ± 0.001	2.28 ± 0.032	2.32 ± 0.31
	Female	107.92 ± 0.93	-	0.0098 ± 0.001	2.28 ± 0.032	2.37 ± 0.27
Janoschek	Male	113.96 ± 0.82	-	0.01 ± 0.001	2.28 ± 0.032	2.32 ± 0.31
	Female	110.29 ± 0.81	-	0.0098 ± 0.001	2.28 ± 0.032	2.37 ± 0.27
Gompertz	Male	125.83 ± 0.94	5.12 ± 0.084	0.30 ± 0.004	-	-
	Female	122.89 ± 0.91	5.06 ± 0.073	0.29 ± 0.004	-	-
Logistic	Male	111.62 ± 0.49	30.52 ± 0.89	0.55 ± 0.006	-	-
	Female	107.53 ± 0.457	30.69 ± 0.804	0.54 ± 0.005	-	-
Lopez	Male	137.18 ± 1.85	7.16 ± 0.085	2.64 ± 0.05	-	2.84 ± 0.32
	Female	134.24 ± 1.799	7.42 ± 0.087	2.62 ± 0.046	-	2.83 ± 0.28
Richards	Male	125.83 ± 1.04	0.0002 ± 0.004	0.30 ± 0.004	− 0.00003± 0.001	-
	Female	122.88 ± 1.04	0.0001 ± 0.004	0.29 ± 0.005	− 0.0005± 0.018	-
Weibull	Male	113.96 ± 0.82	111.64 ± 0.974	0.01 ± 0.001	2.28 ± 0.032	-
	Female	110.29 ± 0.8	107.92 ± 0.933	0.0098 ± 0.001	2.28 ± 0.032	-

BW_0_ = initial body weight (g); α = upper asymptotic body weight (g); t = age (months);

β, k, and m = parameters specific for the function; β characterizes the first part of growth before the point of inflection; k describes the second part in which growth rate decreases until the animal reaches the upper asymptotic body weight or mature body weight (α), m is the shape parameter determining the position of the curve point inflection.

**Table 3:** Akaike information criterion (AIC), Bayesian information criterion (BIC), standard error (SER), coefficient of determination, and correlation coefficient of growth functions for ostriches

Functions	Sex	AIC	BIC	**S**E**R**	Cor	R^2^ (%)
von Bertalanffy	Male	9656.07	9677.47	2.39	0.990	98.11
	Female	11799.78	11822.02	2.19	0.990	98.04
Bridges	Male	9486.51	9513.25	1.39	0.991	98.30
	Female	11603.48	11631.28	1.19	0.991	98.23
Janoschek	Male	9486.51	9513.25	1.39	0.991	98.30
	Female	11603.48	11631.28	1.19	0.991	98.23
Gompertz	Male	9531.22	9552.62	1.67	0.991	98.25
	Female	11647.97	11670.21	1.45	0.991	98.19
Logistic	Male	9552.35	9573.75	1.79	0.991	98.23
	Female	11665.72	11687.96	1.52	0.991	98.17
Lopez	Male	9562.37	9589.11	1.96	0.991	98.22
	Female	11680.47	11708.28	1.70	0.991	98.16
Richards	Male	9533.23	9559.98	1.67	0.991	98.25
	Female	11649.94	11677.74	1.45	0.991	98.19
Weibull	Male	9486.51	9513.25	1.32	0.991	98.30
	Female	11603.48	11631.28	1.13	0.991	98.23

*AIC* Akaike’s information criterion, *BIC* Bayesian information criterion, *Cor* Pearson’s correlation between predicted and actual body weights. *SER* Standard error of the regresion, R^2^ Coefficient of determination

The age and body weights were estimated at different growth phases of ostrich (Table 4). In addition, the body weight, monthly gain (MG), and average monthly gain (AMG) every month from birth to 12 months of age were estimated using the best model among tested models herein were also predicted (Table 5). The predicted body weights (BWs) of the ostriches were calculated using the predict() function in R software, and then Pearson’s correlation (cor) was calculated between the predicted BW and measured BW using the cor() function in the R software. The absolute growth rate (monthly gain, MG_t_ = BW_t_ – BW_t-1_), and average monthly gain ((AMG_t_ = (BW_t_ – BW_0_)/t, in which, BW_t_ is the body weight at t months of age, and BW_0_ is the weight of the bird at birth) were calculated, then the most appropriate slaughter age was determined when the average monthly gain (AMG_t_) was maximum (Bo et al. 2023).

**Table 4:** Estimated age and weight at different growth phases of Ostrich

Functions	Sex	Start of growth acceleration phase^1^		Inflection point^2^		End of growth deceleration phase^3^	
		Age (months)	Weight (kg)	Age (months)	Weight (kg)	Age (months)	Weight (kg)
von Bertalanffy	Male	2.80	13.80	4.95	40.87	15.25	124.16
	Female	2.87	13.66	5.16	40.47	16.08	122.93
Janoschek	Male	2.51	11.40	5.74	48.97	10.62	102.57
	Female	2.56	11.03	5.89	47.41	10.89	99.26
Gompertz	Male	2.65	12.58	5.41	46.29	12.87	113.25
	Female	2.71	12.29	5.59	45.21	13.35	110.60
Logistic	Male	2.22	11.16	6.20	55.81	10.19	100.46
	Female	2.27	10.75	6.33	53.77	10.40	96.78
Lopez	Male	2.85	13.72	5.29	44.52	16.33	123.46
	Female	2.92	13.42	5.45	43.42	17.02	120.81
Richards	Male	2.65	12.58	5.41	46.29	12.87	113.25
	Female	2.71	12.29	5.59	45.21	13.34	110.59

^1^Age at which the ostrich attains 10% of its final body weight and represents the beginning of the growth acceleration phase.

^2^Represents the end of the growth acceleration phase and the beginning of the deceleration phase.

^3^Age at which the ostrich attains 90% of its final body weight and represents the end of the growth deceleration phase.

Note: The inflection point cannot be determined by using Bridges and Weibull functions.

**Table 5:** Actual and estimated body weight (kg), monthly gain (MG, kg/month) and average monthly gain (AMG, kg/month) by Weibull function in commercial ostriches

Month	Male							Female						
	Measured value				Weibull model			Measured value				Weibull model		
	*n*	BW	MG	AMG	BW	MG	AMG	n	BW	MG	AMG	BW	MG	AMG
0	124	0.88 ± 0.09	-	-	2.32	-	-	158	0.87 ± 0.10	-	-	2.37	-	-
1	124	4.11 ± 0.55	3.24	3.24	3.48	1.16	1.16	155	3.99 ± 0.55	3.12	3.12	3.42	1.05	1.05
2	123	8.28 ± 1.29	4.17	3.70	7.84	4.36	2.76	153	8.26 ± 1.29	4.27	3.69	7.39	3.97	2.51
3	122	16.46 ± 2.10	8.17	5.19	15.72	7.88	4.47	153	15.12 ± 2.08	6.86	4.75	14.60	7.20	4.08
4	120	27.19 ± 3.09	10.73	6.58	26.70	10.98	6.09	151	25.10 ± 3.07	9.97	6.06	24.71	10.11	5.58
5	120	39.75 ± 4.48	12.56	7.78	39.85	13.15	7.51	151	36.61 ± 4.45	11.51	7.15	36.92	12.22	6.91
6	120	53.27 ± 5.12	13.52	8.73	53.96	14.11	8.61	150	49.47 ± 5.17	12.86	8.10	50.17	13.25	7.97
7	117	66.83 ± 5.63	13.56	9.42	67.76	13.81	9.35	145	62.92 ± 5.58	13.45	8.86	63.31	13.14	8.71
8	117	79.62 ± 6.29	12.79	9.84	80.22	12.45	9.74	141	74.87 ± 6.16	11.96	9.25	75.36	12.05	9.12
9	117	91.07 ± 6.47	11.45	10.02	90.62	10.40	9.81	141	85.89 ± 6.38	11.01	9.45	85.62	10.26	9.25
10	117	101.81 ± 6.66	10.75	10.09	98.70	8.08	9.64	141	96.32 ± 6.63	10.43	9.54	93.78	8.15	9.14
11	117	103.26 ± 6.81	1.45	9.31	104.55	5.85	9.29	141	98.72 ± 6.62	2.41	8.90	99.83	6.05	8.86
12	117	107.68 ± 7.90	4.42	8.90	108.49	3.94	8.85	141	103.43 ± 7.57	4.70	8.55	104.02	4.20	8.47

## Results

### Parameters of the growth curve

The growth performance of male ostriches was higher than females, so mature asymptotic body weight (α) of the male ostriches was higher than that of the female ostriches in all models (Table 2). This α value was the highest in the von Bertalanffy function and was the lowest in the Logistic function for both male and female ostriches (Table 2). The mature asymptotic body weight of male ostriches was estimated from 111.62 kg (Logistic) to 137.95 kg (von Bertalanffy) and this value in female ostriches ranged from 107.53 kg (Logistic) to 136,58 kg (von Bertalanffy) (Table 2).

The estimated mature growth rate (k) represents growth ability in the second phase and this value would be gradually decreased until the animal reached mature weight. In this study, the k values of male ostriches that were estimated by Bertalanffy, Gompertz, Logistic, Lopez, and Richards functions were higher than those of female birds. The k values that were estimated by Bridges, Janoschek, and Weibull functions were the same for both male and female ostriches. The k value that was estimated by the Lopez function was the highest.

### The best non-linear model for describing the growth curve of ostriches

The important criteria to evaluate a fitted model of the growth curve were R^2^, AIC, BIC, and SER. The highest R^2^ value and the lowest AIC, BIC, and SE values indicate a better model. The R^2^ was very high in all models (R^2^ > 98%), and the Pearson’s correlations between predicted and actual body weight were very high (cor = 0.99 in all models) (Table 3). This information suggests that eight tested models are suitable to describe the growth curves of ostriches reared in tropical conditions in Vietnam.

To choose the correct and better model, the BIC, AIC, and SER were ranked from low to high (Table 3). The better model was confirmed if the function had the lowest values of BIC, AIC, and SER. Thus, the Weibull function was the best model among 8 tested models that describes the growth rate of both male and female ostriches with the highest coefficient of determination (R^2^ = 98.30, and 98,23 %), the lowest AIC (AIC = 9,486.51, and 11,603.48), the lowest BIC values (BIC = 9,513.25, and 11,631.28), and the lowest SER values (SER = 1.32, and 1.13) (Table 3).

In addition, the results from Table 3 also show that the Bertalanffy function was the worst model to describe the growth rate of ostriches in Vietnam because this function had the lowest R^2^ and the highest AIC, BIC, and SER values in both male and female ostrich (Table 3).

However, it should also be noted that the growth data of ostriches in this study were measured up to 12 months of age, not until the final body weight when the ostriches can reach a fully mature body weight. Thus, the appropriateness of the above functions for ostriches in this study was only guaranteed within 12 months of age.

### The growth phases

The estimated ages of male ostriches were lower than those of female birds at all growth phases and in all models (Table 4). The age at the start of the growth acceleration phase of male ostriches was estimated from 2.22 to 2.85 months. Whereas this figure for female ostriches was later, from 2.27 to 2.92 months. Similarly, the estimated age at the inflection point of male ostriches (4.95 to 6.20 months) was lower than female ostriches (5.16 to 6.33 months). Consequently, the age of male ostriches at the end of the growth deceleration phase was predicted from 10.19 to 16.33 months, while that of the female ostrich was from 10.40 to 17.02 months. In addition, the estimated weight for female ostriches at all growth phases was lower than those of males in all the models (Table 4).

### The suitable slaughter ages

**Fig. 1:**
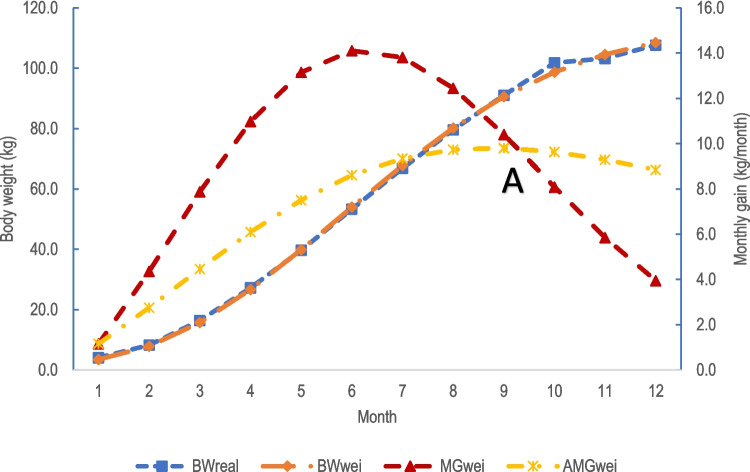
The actual and estimated growth curves of male ostriches raised in tropical condition in Vietnam. Note: BWreal = the actual body weight; BWwei = estimated body weight using Weibull function; MGwei = estimated monthly weight gain using Weibull function; AMGwei = estimated average weight gain using Weibull function. The suitable slaughter age of male ostriches was at the cutting point A (between MGwei and AMGwei), and estimated slaughter weight for male ostriches was 93 kg

**Fig. 2:**
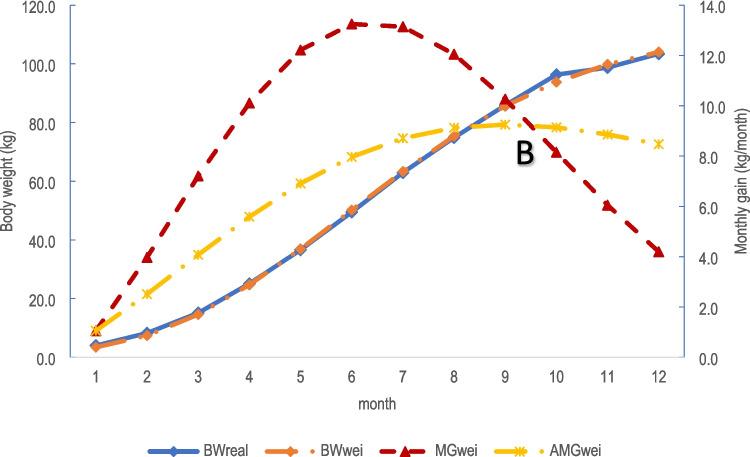
The actual and estimated growth curves of female ostriches raised in tropical condition in Vietnam. Note: BWreal = the actual body weight; BWwei = estimated body weight using Weibull function; MGwei = estimated monthly weight gain using Weibull function; AMGwei = estimated average weight gain using Weibull function. The suitable slaughter age of female ostriches was at the cutting point B (between MGwei and AMGwei), and the estimated slaughter weight for female ostriches was 90 kg

The results of estimated body weight by month, monthly gain, and average monthly gain by Weibull function for male and female fattening ostriches were compared with measured data and presented in Table 5, Figs. 1 and 2. The BW of ostriches at the 12 months of age was 107.68 kg and 103.43 kg for male and female ostriches, respectively. The monthly gains reached the maximum level was estimated using Weibull function at 6 months of age for both male ostriches (14.11 kg) and female birds (13.25 kg) (Table 5). The average monthly gain using Weibull model was at the highest level at 9 months of age for both male (9.81 kg/month) and female ostriches (9.25 kg/month) (Table 5). The maximum average monthly gains were obtained between 9 and 10 months of age for both male and female ostriches, and these times were identified as the best time for slaughtering. The suitable slaughter ages were also confirmed in Figs. 1 and 2, where the cutting point was created between the MG curve and the AMG curve (Figs. 1 and 2). At this suitable slaughter time, the weights of ostriches were estimated at 93.00 kg for males (at 9.27 months of age) and 90.00 kg for females (at 9.48 months of age).

The curves that described measured BW and predicted BW overlapped in both genders (Figs. 1, and 2). Similar values of measured BW and predicted BW indicated that the Weibull function was the best model among tested models to describe the growth of male and female ostriches in this study.

## Discussion

### The models for describing the growth curve of ostriches

Eight growth functions tested in this study could be used for modeling the growth curve of fattening ostriches raised in tropical conditions, because of high R^2^ and high correlations between measured BWs and predicted BWs values from all these models. However, the Weibull function was the best model among these 8 tested models for both male and female ostriches because this Weibull model generated the highest R^2^ value (R^2^ = 98.30, and 98.23%) and the lowest AIC and BIC values compared to the other models (Table 3).

The upper asymptotic body weight (α) of the Weibull function in ostriches was in the range of reported in Spain (Faridi et al. 2015) and in Brazil (de Rezende et al. 2021; Ramos et al. 2013). In this study, α values were estimated using eight functions range from 112 to 138 kg for males, and from 108 to 137 kg for females (Table 2). This mature BW of the ostriches in Vietnam was in the range of previous studies. The mature BW was estimated from 102 to 125 kg (for males), and 98 to 122 kg (for females) (Cilliers et al. 1995; Du Preez et al. 1992; Ramos et al. 2013). However, if using the Weibull function (best fit function), α were estimated at 113.96 kg for males and 110.29 kg for females. The α values using best fit function (Weibull) herein were in the range of previous studies, 110.40 kg for ostriches raised in Spain using the Richard function (Faridi et al. 2015), 115.5 kg to 129.5 kg for male ostriches and 137.3 kg to 145.2 kg for female ostriches using Gompertz model used to estimate the growth of ostriches raised in Brazil (de Rezende et al. 2021). The little differences between the above research results may be due to the influence of growth factors such as nutrition, breeds, and management.

The coefficient of determination (R^2^) from all eight models herein (R^2^ = 98.04 to 98.30) was lower than those in the study by Faridi et al. (2015) using the Richard function (R^2^ = 99.5 to 99.7). However, the R^2^ from eight functions in this study was higher than those reported by Ramos et al. (2013) (R^2^ = 92.4 to 93.8).

The maturation rate (growth rate k) in this study described the second part the growth rate which decreased until ostriches reach the mature body weight. The k value observed with the best function (Weibull) for the ostriches in this study was similar between male and female ostriches. These figures were 0.01 kg per month for both male and female ostriches (Table 2). These values were higher than those reported in ostriches raised in Brazil (k = 0.0071 to 0.0088 kg per day for females; k = 0.0066 to 0.0076 for males) (de Rezende et al. 2021), and ostriches raised in Spain (k = 0.009 to 0.019 kg per day for females, and k = 0.008 to 0.018 kg per day for males) (Ramos et al. 2013). The Weibull function fits the relative growth rate of ducks (Vitezica et al. 2010).

### The suitable slaughter ages

The estimated age (4.95 to 6.33 months) and body weight (40.47 to 55.81 kg) at the inflection point of ostriches in this study (Table 4) were in the range of estimated age and body weight reported by de Rezende et al. (2021), (Faridi et al. 2015), Ramos et al. (2013). The African black ostrich, red neck ostrich, and crossbred between these two breeds had an age at the inflection point from 161.72 to 198.38 days for female ostriches, and the estimated body weight from 42.45 to 47.64 kg; whereas these values for male ostriches were 180.75 to 208.52 days and the estimated body weight from 50.51 to 52.75 kg (de Rezende et al. 2021). In a study by Ramos et al. (2013), the ages at the inflection point of ostriches raised in Brazil were from 169.66 to 185.06 days for female ostriches, with estimated body weight from 44.28 to 50.85 kg; these figures for male ostriches were 173.84 to 188.74 days and estimated body weight from 45.97 to 51.83 kg. However, the results reported by Faridi et al. (2015) were very low, the results showed that ostriches raised in Spain had aged at the inflection point of 16.1 weeks (112.7 days), and body weight at the inflection point of 27.8 kg. Some reasons for the differences among the studies are possibly diets, housing, breeding, managements, methods used for evaluation. The lower age at the inflection point of ostriches in Spain by Faridi et al. (2015) compared to our study because Faridi et al. (2015) used three approaches to describe growth performance of male and female ostriches separately.

The appropriate growth function not only allows the estimate of the body weight of the ostrich but also helps to estimate the rate of body weight gain at any point in the breeding cycle as well as average body weight gain up to those dates. The results herein (Table 5, Figs. 1 and 2) showed that the estimated body weight was very consistent with the measured body weight in both male and female ostriches. This suggests that the growth function (Weibull function) in this study was used to estimate the growth of ostriches raised in tropical conditions accurately. This also indicates that it was not necessary to weigh the body weight of fattening ostriches at different ages, we can estimate its body weight using these functions with high accuracy. An accurate estimation of both body weight and growth rate over age as above will allow us to calculate the nutrition requirement for body maintenance (by body weight) and for growth (by growth rate) and then can determine the appropriate diet for ostriches at each age stage. That was one of the practical implications of determining the specific growing function of ostriches in this study. However, as discussed above, the growing function determined for ostriches in this study was only valid for certain applications within 12 months of age because the older age has not been investigated.

Results based on measured data and estimated growth function show that the average body weight gain for the whole period up to month t (AMGt) also increased gradually in the first months of age, reaching the maximum at 9 months of age, and then gradually decreased. However, the varied rate of AMGt was slower than that of MGt; when MGt was at its maximum (at the inflection point of the BWt graph), then AMGt was still increased but lower than MGt until AMGt peaked at the point of AMGt = MGt. The maximum value of AMGt was lower than the maximum value of MGt, but after AMGt reached the maximum, AMGt > MGt.

Similar values of measured BW and predicted BW using the Weibull model (Table 5), and the overlapped curves between these two values confirmed that Weibull was the best model herein to report the growth of ostriches. The maximum AMGt value was highest at 9 and 10 months of age for both male ostriches and female ostriches (Table 5) suggesting that 10 months of age was the best slaughter time. In addition, this the best slaughter age also can be seen in the Figs. 1 and 2, where AMGt = MGt (the point where two curves (AMGt and MGt) were cut in Figs. 1 and 2). The results show that AMGt peaked at 9.27 months of age for males (the estimated body weight of 93 kg), and 9.50 months of age for females (the estimated body weight of 90 kg). Therefore, the ages for the above estimated maximum AMG were considered technically optimal slaughter ages for male and female ostriches. The results reported by de Rezende et al. (2021) illustrated that the slaughter age for all three kinds of ostriches (African black ostrich, red neck ostrich, and crossbred between these two breeds) was about 390 days of age (about 13 months of age).

## Conclusions

The Weibull was the best function among 8 tested models for modeling the growth of both male and female ostriches raised in tropical conditions. By using the most accurate model, Weibull, the estimated slaughter ages for male and female ostriches were suggested from 9 to 10 months of age, and the estimated weights were 93 kg for males (at 9.27 months of age) and 90.00 kg for females (at 9.48 months of age). Thus, these eight mathematical functions, especially the Weibull model can be used to estimate the growth of ostriches in Vietnam and other tropical countries.

## Data Availability

The data of this study will be shared upon reasonable request to the corresponding author.

## Abbreviations

AMG: Average monthly gain
AIC: Akaike’s information criterion
BIC: Bayesian information criterion
BW: Body weight
R^2^: Coefficient of determination
MG: Monthly gain
SER: Standard error of the regression
